# Migration of a Swallowed Blunt Foreign Body to the Neck

**DOI:** 10.1155/2014/646785

**Published:** 2014-01-30

**Authors:** Kerem Ozturk, Goksel Turhal, Sercan Gode, Atilla Yavuzer

**Affiliations:** Department of Otolaryngology, Ege University School of Medicine, Izmir, Turkey

## Abstract

Ingestion of foreign bodies is a common problem in the otolaryngology practice. Reports of extraluminal migration of the foreign bodies from the upper aerodigestive tract are rare. Penetration and extraluminal migration of ingested foreign bodies may cause severe vascular and suppurative complications, even death. We report a 4-year-old girl who presented with a mass and partial extrusion of a foreign body in the neck. She had a history of ingesting the plastic top piece of a knitting needle approximately 1 year ago. She had been asymptomatic until the present time. The examination revealed a red, blunt, rectangular plastic foreign body half embedded in the skin of the right neck. Esophagography with barium swallow, cervical X-rays, and computed tomography scans were obtained. The foreign body was easily removed under general anesthesia. Primary closure and direct laryngoscopy was also performed. The patient recovered very well without any complications.

## 1. Introduction

Foreign body ingestion is a common problem among pediatric populations. Most of the ingested foreign bodies pass naturally through the gastrointestinal tract, but 10–20% require nonoperative intervention and 1% or less require surgery [1]. Even though there are numerous case reports of ingested foreign bodies of the upper aerodigestive tract, only a small number perforate the esophagus and an even smaller fraction migrate extraluminally. Most of these migratory foreign bodies reported so far have been fish bones and other sharp objects. The case described here is a report of an ingested blunt, plastic foreign body migrating into the neck. This case highlights the possibility that relatively big blunt foreign bodies can also penetrate the mucosa, migrate extraluminally, and present as a foreign body in the neck.

## 2. Case Report

**Figure 1:**
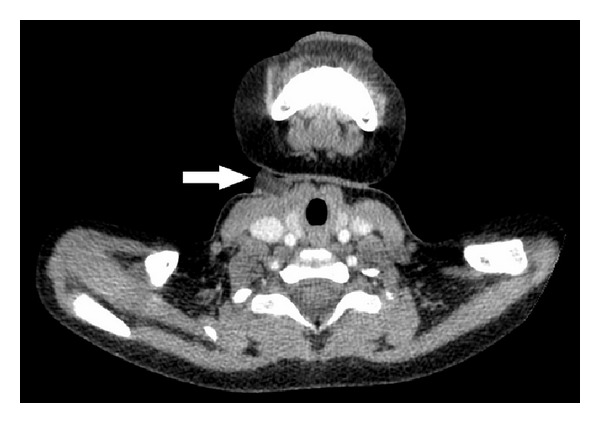
Computed tomography section that shows the foreign body in the right side of the neck (arrow).

A 4-year-old girl was referred to our institution with a mass and partial extrusion of a foreign body in the neck. Symptoms started 4 days ago with a redness and swelling on the right neck, followed by partial extrusion of a red, blunt foreign body. A computed tomography (CT) scan revealed a foreign body with prominent margins anterior to sternocleidomastoid and infrahyoid muscles, half being embedded in the skin and the other half being outside (Figure 1). No fistula was found with the deeper tissues. The family remembered that the girl swallowed the plastic top piece of a knitting needle approximately one year ago. She had been asymptomatic until the present time.

Physical examination revealed a partially extruded red, plastic foreign body about 1 cm diameter in the right neck. The patient did not have fever. There was no any tenderness or pain in the affected region. The rest of the head and neck examination did not reveal any abnormalities. A blood sample examination revealed a normal white blood cell count (7,640/mm^3^) and a slightly elevated C-reactive protein level (2.2 mg/dL).

**Figure 2:**
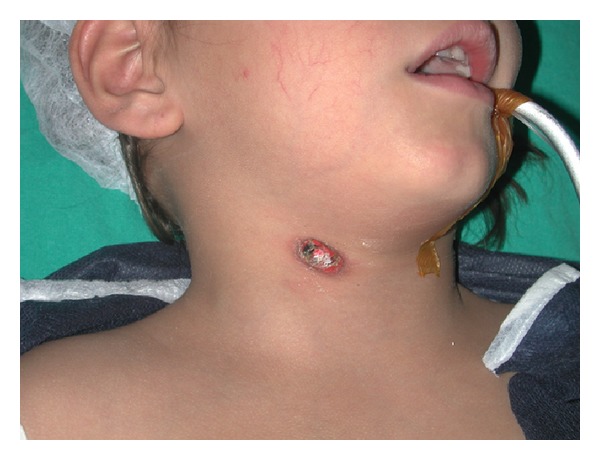
Foreign body in the right side of the neck before removal.

**Figure 3:**
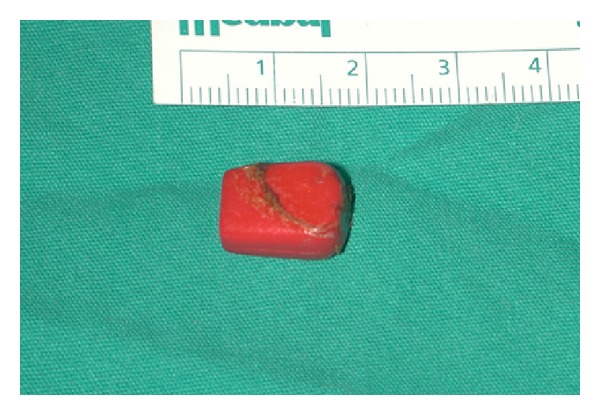
The foreign body after removal.

The patient was hospitalized after the initial examination and CT scan. An oral prophylactic antibiotic was administered (Clarithromycin 500 mg/day). The foreign body was held only with skin and subcutaneous fatty tissue. Also lateral neck X-rays and esophagography with barium swallow were obtained. Esophagography did not reveal any fistula tract between the foreign body and the upper aerodigestive tract. Direct laryngoscopy was performed under general anesthesia the next day. Larynx, cervical esophagus, and pyriform sinuses were found normal. The foreign body was removed easily (Figures 2 and 3). The foreign body had a rectangular shape, was made from hard plastic, and was 13 mm in length. The cavity was filled with granulation tissue. The examination did not reveal any pus or presence of a fistula tract with deeper tissues. Primary suturation was applied to the wound. Postoperatively, the patient recovered very well without any complications.

## 3. Discussion

The incidence of ingested foreign bodies penetrating the esophagus and being extraluminal in the neck is fairly rare. Ingested foreign bodies, especially fish bones, become lodged in the palatine tonsil, the base of tongue, the vallecula, the pyriform sinus, and the esophagus. Due to the direction and site of the migration of the foreign body, severe or even fatal complications may occur. These are aortoesophageal fistula, innominate esophageal fistula, subclavian esophageal fistula, carotid rupture, and local suppurative processes, such as periesophageal abscess, mediastinitis, retropharyngeal abscess, thyroid abscess, and deep neck abscess [2–4].

In our case the ingested foreign body might have become lodged in the right pyriform sinus of the hypopharynx or in the cervical esophagus, penetrated and passed through the mucosa which healed over later, and migrated out of the neck after silent period of 1 year. The plastic, blunt structure and smooth surface of the foreign body might have protected the patient from vascular and suppurative complications. This case report is the first to describe migration of blunt foreign bodies in the neck. Patients with foreign body aspiration or swallowing should be closely followed up.

Besides anamnesis and examination radiology is an important tool to detect foreign bodies and their location. CT scan can be effectively used to locate foreign bodies [5, 6]. A mass or an abscess in the head and neck region could be an outcome of a migrated foreign body. Therefore a thorough history must be obtained and an ingestion of a foreign body must be questioned.
